# Nature-based physical activity in breast cancer survivors: a new frontier in supportive care

**DOI:** 10.3389/or.2026.1837259

**Published:** 2026-07-08

**Authors:** Giuditta Carretti, Mirko Manetti, Mirca Marini

**Affiliations:** Section of Anatomy and Histology, Department of Experimental and Clinical Medicine, University of Florence, Florence, Italy

**Keywords:** breast cancer survivorship, exercise oncology, green prescription, nature-based interventions, outdoor physical activity, psychophysiological health, quality of life, supportive cancer care

## Abstract

Breast cancer (BC) survivors frequently experience long-term physical and psychosocial sequelae following treatment, including cancer-related fatigue, cardiovascular dysfunction, sarcopenia, sleep disturbances, and emotional distress, which can significantly affect quality of life. Structured physical activity is widely recognized as a key component of supportive oncology care and has demonstrated benefits in improving physical function, psychological wellbeing, and survival outcomes. However, adherence to exercise recommendations remains suboptimal in cancer patients mostly due to treatment-related symptoms, motivational barriers, and limited access to tailored supervised programs. Though the field remains relatively underexplored within exercise oncology, nature-based physical activity (NBPA), defined as exercise performed in natural environments, is emerging as a promising complementary approach that integrates the physiological benefits of physical exercise with the restorative effects of nature exposure. Growing evidence indicates that natural settings can support stress reduction, autonomic regulation, immune function, and psychological resilience, potentially enhancing the overall benefits of physical activity. Several effects have been documented in broader populations and, to a lesser degree, among cancer survivors, while direct evidence specifically addressing BC survivors remains limited. Therefore, many of the proposed psychophysiological mechanisms should currently be regarded as plausible and biologically supported hypotheses requiring further validation through dedicated clinical studies. This perspective paper synthesizes current evidence on the role of NBPA in BC survivorship by discussing the psychophysiological mechanisms underlying health effects of nature exposure in this vulnerable population and the main investigated outdoor activities, including walking and Nordic walking, aquatic disciplines, green mind-body practices, and forest-based programs. Finally, the article highlights research gaps and future directions aiming at providing concrete hints to stably integrate nature-based interventions into multidisciplinary survivorship care pathways addressing BC survivors.

## 1 Introduction

Breast cancer (BC) is the most frequently diagnosed malignancy among women worldwide. Although advances in screening and treatment have substantially improved survival rates (1–4), many survivors experience persistent physical and psychosocial complications, including cancer-related fatigue, cardiotoxicity, sarcopenia, lymphedema, sleep disturbances, cognitive impairment, anxiety, and depression (5–8). These conditions can jeopardize daily functioning and quality of life and are often linked to shared biological mechanisms such as chronic inflammation, autonomic imbalance, and neuroendocrine dysregulation (9). Consequently, improving long-term supportive care has become a priority in modern oncology.

Among non-pharmacological strategies, structured exercise is widely recognized as a cornerstone of supportive cancer care (3,10–12). Compelling evidence demonstrated that adapted physical activity can reduce treatment-related side effects while improving cardiorespiratory fitness, muscular strength, psychological wellbeing, and survival outcomes (5,13–18). Based on this growing evidence and interest, the American College of Sports Medicine “Moving Through Cancer” initiative has formalized exercise recommendations for individuals living with and beyond cancer (12,19). However, despite established guidelines, adherence to recommended physical activity levels remains suboptimal among BC survivors (20–22) mostly due to treatment-related symptoms, logistical barriers, and limited access to tailored programs (23–26). Traditional exercise prescription in oncology is primarily based on physiological parameters such as intensity, frequency, and duration, often overlooking contextual factors that influence motivation and long-term engagement (27). Behavioral research indicates that enjoyment and intrinsic motivation are critical determinants of sustained physical activity, suggesting that environmental context may play an important role in adherence.

Nature-based physical activity (NBPA), defined as exercise performed in natural environments, has emerged as a promising approach that combines the physiological benefits of exercise with the restorative effects of nature exposure (28–33). Contact with natural settings has been associated with improved stress regulation, autonomic function, immune responses, and psychological wellbeing (34–38), which are mechanisms particularly relevant for BC survivorship. Natural environments may also increase enjoyment and reduce perceived exertion during exercise, potentially supporting long-term adherence (39–41). In the present article, NBPA is broadly conceptualized as a continuum of interventions combining exposure to natural environments with bodily movement. This continuum includes both structured exercise modalities performed outdoors (e.g., walking, Nordic walking, and aquatic activities) and activities characterized by lower physical demands but substantial engagement with natural environments (e.g., forest bathing and outdoor mind-body practices). While these approaches differ considerably in exercise intensity, supervision, physiological load, and therapeutic objectives, they share the fundamental feature of integrating nature exposure into supportive care experiences.

Despite the aforementioned potential benefits, the integration of nature exposure within structured exercise interventions in oncology remains limited. On this basis and leveraging the expertise of our research group in the field of adapted kinesiology, the present perspective article explores NBPA as a promising integration of exercise prescription for BC survivors. Through a narrative appraisal of the literature on exercise oncology, nature exposure, green exercise, forest medicine, and supportive cancer care, we summarized current evidence, discussed potential mechanisms, and identified key research priorities, with the main objective of synthesizing representative and clinically relevant findings to guide future research interventions. Ultimately, this perspective article aims to provide actionable insights to concretely integrate nature-based interventions into supportive oncology care for this vulnerable population.

## 2 Evidence-based benefits of structured physical activity in breast cancer survivors

Structured physical activity represents one of the most effective non-pharmacological strategies for mitigating treatment-related side effects and improving survivorship outcomes in BC population (17,21,42,43). Extensive research has demonstrated that regular exercise improves cardiorespiratory fitness, muscular strength, body composition, and physical functioning in BC survivors (44,45). Exercise has also been associated with reductions in cancer-related fatigue, one of the most common and debilitating symptoms experienced after cancer treatment (46–48). Additional benefits include improvements in sleep quality, psychological wellbeing, and overall health-related quality of life (45,49–51). Beyond symptomatic relief, exercise may influence biological pathways involved in cancer progression and recovery. Physical activity modulates inflammatory processes, oxidative stress, insulin signaling, and immune function, which are all mechanisms associated with improved health outcomes in cancer populations (52,53). Exercise-induced myokines released during muscle contraction have also been suggested to play a key role in regulating tumor growth and cellular metabolism (53–55). Epidemiological studies indicated that higher levels of physical activity following diagnosis are associated with improved survival outcomes. In particular, evidence from large prospective cohorts suggested that regular post-diagnosis exercise is linked to lower BC-specific and all-cause mortality (13,56–58). Similarly, structured exercise programs implemented during or after treatment have demonstrated beneficial effects on cardiovascular fitness and cardiac function, which are particularly relevant given the cardiotoxic effects of some oncologic therapies (59–61).

Despite these well-documented benefits, many BC survivors struggle to maintain regular exercise participation due to treatment-related symptoms, fear of injury or lymphedema exacerbation, psychological distress, and limited motivation (20,62). These barriers highlight the need for innovative approaches capable of enhancing enjoyment, motivation, and long-term adherence to physical activity within non medicalized frames (27,63,64). In this perspective, NBPA may represent a promising emerging strategy that combines the physiological benefits of exercise with the restorative effects of nature exposure. The potential added value of NBPA seems to reside less in replacing established exercise prescriptions and more in enhancing behavioral engagement, perceived enjoyment, sustainability of participation, and stress reduction. Indeed, preliminary comparative studies suggest that natural environments may amplify some psychological benefits of exercise (28,30,31). However, robust evidence demonstrating superior clinical outcomes compared with indoor exercise remains limited, and further targeted investigations are needed.

## 3 Psychophysiological effects of nature exposure for breast cancer survivorship

Nature exposure has been increasingly recognized as an important determinant of human health and wellbeing. In fact, time spent in natural environments such as forests, parks, gardens, or coastal areas has been associated with improvements in mental health, stress reduction, and overall wellbeing across diverse populations (28,65,66). The concept of “green exercise”, referring to physical activity performed in natural settings, has gained attention as a multidimensional approach that integrates environmental exposure with exercise physiology, hence potentially amplifying the benefits of traditional interventions (41,67). Two major theoretical frameworks help explain the positive effects of nature exposure on human health. The Attention Restoration Theory proposes that natural environments facilitate recovery from mental fatigue by engaging effortless attention processes (68,69), while the Stress Reduction Theory suggests that exposure to natural landscapes triggers innate psychophysiological responses able to promote relaxation and reduce stress (70–72). Empirical research supports these models, demonstrating improvements in autonomic regulation and reductions in hypothalamic-pituitary-adrenal axis activation following nature exposure, reflected in lower cortisol levels, decreased blood pressure, and improved heart rate variability (73–75). Evidence also indicates that nature exposure may influence immune and inflammatory pathways relevant to cancer survivorship, with studies conducted in forest environments reporting increased activity of natural killer cells and enhanced expression of anticancer proteins following such immersive experiences (35,76–78). Notably, several lines of evidence regarding autonomic regulation, neuroendocrine and inflammatory modulation, and immune enhancement mostly derive from studies conducted in healthy individuals or heterogeneous populations (35,78). Although preliminary investigations involving BC survivors have reported encouraging findings (76,79,80), direct mechanistic evidence specifically demonstrating such effects in this target population remains relatively scarce. Consequently, the biological pathways herein highlighted should be interpreted as plausible explanatory frameworks rather than established causal mechanisms. Collectively, these findings suggest that nature exposure may contribute to improved immune surveillance and modulation of inflammatory processes. Exercise further stimulates the release of myokines such as interleukin-6, irisin, and myonectin, which exert systemic anti-inflammatory and immunomodulatory effects and may influence tumor biology (53,55). When exercise is performed in natural environments, these biological responses may occur alongside the stress-reducing and immune-modulating effects of nature exposure, potentially generating additive or synergistic benefits (81,82).

Psychological outcomes are also strongly influenced by nature exposure. Outdoor environments have been shown to improve mood, reduce symptoms of anxiety and depression, and increase feelings of vitality and social connectedness (83–85). Natural settings may facilitate mindfulness, sensory engagement, and meaningful social interaction, contributing to emotional regulation and psychological resilience (41,67,71). Indeed, systematic reviews have shown that green exercise leads to greater improvements in emotional wellbeing and perceived energy compared with indoor exercise modalities (66,83,86). In addition, qualitative studies demonstrated that natural environments could foster a sense of purpose, empowerment, and reconnection with the body during the recovery process (87–89). For BC survivors, who frequently experience chronic stress and emotional distress following diagnosis and treatment, these benefits may be particularly meaningful (7,90,91).

**FIGURE 1:**
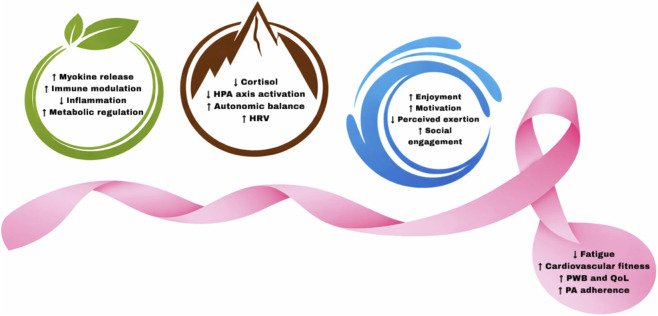
Multidimensional integrated pathways underlying the benefits of nature-based physical activity in breast cancer survivorship. HPA, hypothalamic-pituitary-adrenal; HRV, heart rate variability; PWB, psychological well-being; QoL, quality of life; PA, physical activity.

On the basis of the aforementioned evidence, NBPA interventions may therefore offer a holistic approach that addresses both physical and psychological dimensions of recovery while fostering greater adherence to physical activity and healthier lifestyle behaviors among BC survivors (20,62,92–94). The conceptual framework through which NBPA may influence BC survivorship outcomes by integrating biological, psychophysiological, and behavioral pathways is graphically illustrated in Figure 1.

## 4 Main nature-based physical activities investigated in breast cancer survivors

Although research on NBPA in oncology is still emerging, several outdoor exercise modalities have been explored in BC survivorship. These activities, though varying in intensity, structure, and therapeutic goals, share the common feature of being conducted in natural or outdoor environments. For conceptual clarity, two partially overlapping frames/modalities have been considered and analyzed. Specifically, exercise-dominant NBPA modalities (i.e., walking, Nordic walking, and aquatic activities), in which physical training constitutes the primary therapeutic component, and nature-exposure-dominant interventions (i.e., forest bathing and outdoor mind-body practices), in which environmental immersion and sensory engagement represent the principal elements while physical exertion remains secondary, were evaluated. Such an integrated perspective may help contextualize the heterogeneous evidence currently available while providing a comprehensive overview. Notably, the strength of evidence varies considerably across NBPA modalities. Indeed, walking, Nordic walking, and dragon boat racing have been directly investigated in BC survivors, while several proposed psychophysiological mechanisms and health effects associated with forest-based interventions and nature exposure derive primarily from studies conducted in healthy individuals or broader clinical populations. The overall features and evidence-based benefits of the main investigated NBPAs specifically addressing individuals living with and beyond BC are detailed in the following paragraphs and schematically summarized in Table 1.

**TABLE 1:** Features and reported benefits of the main nature-based physical activities investigated in breast cancer survivorship.

Nature-based physical activity	Key features and context	Evidence-based benefits	Main references and evidence level
Nature-based walking	Accessible, low-cost, adaptable intensity	↓ Fatigue and perceived stress	(95–100) Direct BC survivor evidence
	Individual or group based	↑ Physical functioning, sleep quality, psychological wellbeing, emotional restoration, and adherence	
	Easily integrated into daily life		
	Performed in parks/green spaces		
Nordic walking	Walking with poles engaging upper body	↓ Perceived pain	(101–106) Direct BC survivor evidence
	Structured/supervised programs performed at moderate intensity	↑ Trunk endurance, postural control and flexibility, upper limb function, and quality of life	
	Suitable for outdoor settings	Potential benefits for lymphedema and arthralgia	
Outdoor aquatic exercise	Low-impact activity performed in lakes or coastal waters	↓ Joint stress and fatigue	(87, 107–109) Mixed cancer survivor evidence
	Buoyancy reduces joint load	↑ Mobility, functional recovery, lymphatic/venous return, and emotional wellbeing	
	Suitable for deconditioned subjects		
Dragon boat	Team-based paddling sport	↓ Inflammation	(110–118) Direct BC survivor evidence
	Moderate-high intensity	↑ Cardiovascular fitness, muscle strength, upper-body function, social support, and psychological wellbeing	
	Strong social component		
	Outdoor water setting (rivers and coastal waters)	Safe for lymphedema	
Sailing	Group-based, skill-oriented activity	↓ Psychological distress	(119–121) Preliminary BC survivor evidence
	Balanced combination of physical effort, teamwork, and marine environment exposure	↑ Quality of life, autonomy, confidence, and connectedness	
Outdoor mind-body practices (yoga, tai chi, qigong)	Low-intensity mindful movement	↓ Stress, anxiety, and depression	(99, 122–124) Mixed cancer survivor evidence
	Breathing exercises and meditation	↑ Relaxation, body awareness, emotional regulation, and sleep quality	
	Performed in natural environments		
Forest-based activities (Shinrin-yoku/forest bathing)	Non-strenuous/light activity	↓ Cortisol and blood pressure	(74, 125–129) Predominantly indirect nature-exposure evidence
	Multisensory engagement	↑ Heart rate variability, immune function, psychological wellbeing, and quality of life	
	Immersive exposure to forest environments		

### 4.1 Walking and nordic walking

Walking is one of the most accessible, safe, and adaptable forms of physical activity for BC survivors and can be easily integrated into daily routines. Walking-based interventions across cancer continuum have demonstrated improvements in fatigue, physical functioning, sleep quality, and psychosocial wellbeing (95–98). Randomized trials also highlighted potential benefits for body composition, immune parameters, and cognitive functioning during chemotherapy (51,130–132). Of note, walking programs are generally feasible and well accepted during and after treatment, with studies reporting good adherence among women undergoing chemotherapy (133,134). When performed in natural environments, walking may provide additional psychological benefits. Nature-based walking interventions have been associated with reduced perceived stress, improved emotional wellbeing, and greater perceived restoration among BC survivors (99,100). Programs that incorporate elements of mindfulness and environmental awareness may further enhance psychophysical engagement by encouraging attention to sensory stimuli provided by nature (135,136). Group-based outdoor walking may also promote social interaction and collective coping, creating supportive spaces where movement, conversation, and natural surroundings contribute to recovery after cancer treatment (89).

Nordic walking, which incorporates the use of poles to engage upper body muscles, represents a variation of walking that may offer additional musculoskeletal benefits. Studies have reported improvements in trunk muscle endurance, posture, flexibility, and upper limb function following Nordic walking programs (101–103,137). Systematic review also indicated improvements in pain perception, physical functioning, and quality of life among BC survivors (104). Furthermore, this discipline is safe and well accepted (138), with preliminary evidence suggesting benefits for aromatase inhibitor-related arthralgia, cancer-related pain (139), and upper-limb complications such as lymphedema (105,106).

Overall, both walking and Nordic walking represent practical and scalable NBPA strategies that can be integrated into survivorship care pathways. Their accessibility, adaptability, and suitability for outdoor environments make them particularly valuable for promoting long-term physical activity while simultaneously harnessing the restorative benefits of nature.

### 4.2 Outdoor aquatic activities

Water-based activities represent another promising exercise modality for BC survivors, particularly for individuals experiencing joint pain, cancer-related fatigue, or mobility limitations. The buoyancy of water reduces mechanical stress on joints while facilitating controlled movement and promoting venous and lymphatic return, which may help manage lymphedema-related symptoms (107,108,140). Aquatic exercise may therefore represent a valid alternative for survivors who encounter barriers to conventional exercise, offering a low-impact environment helpful for gradual reconditioning and symptom management (109). Growing interest in these activities is reflected in recent clinical research exploring structured aquatic exercise programs tailored to BC survivorship and comparing land- and water-based aerobic exercise interventions aimed at clarifying the specific physiological and psychosocial benefits of aquatic training in this population (79,140). When conducted in natural aquatic environments such as lakes or coastal areas, these activities may also provide restorative benefits associated with “blue spaces”, including reduced stress and improved emotional wellbeing (87), factors increasingly recognized as relevant to the holistic recovery process following cancer treatment.

Among aquatic sports, dragon boat racing has become particularly popular within BC communities, representing both a rehabilitation activity and an effective symbol of empowerment. Participation in dragon boat teams has been associated with improvements in cardiovascular fitness, muscular strength, and upper-body function, while also promoting social support and psychological wellbeing (110–116,141). Recent studies have suggested that dragon boat participation may positively influence mood, inflammatory responses, and hormonal profiles, supporting both psychological and physiological recovery processes (142,143). Observational studies have highlighted benefits for quality of life and emotional recovery (117,144). Notably, participation appears to be safe for women with or at risk of BC-related lymphedema, with some investigations indicating that paddling-based activities do not increase, and may even help reduce, the risk of arm swelling (115,118,145).

Similarly, sailing programs have been explored as therapeutic experiences that combine physical activity, teamwork, and immersion in natural marine environments, hence progressively attracting attention as potential supportive interventions for BC survivors. Pilot studies focused on group-based sailing protocols suggested that tailored interventions can reduce psychological distress and improve quality of life among these individuals by promoting autonomy, confidence, and social connectedness (119–121).

Collectively, outdoor aquatic activities represent an emerging and multifaceted area within nature-based supportive care for BC survivors. Despite such evidence and growing interest, further research is needed to better understand the mechanisms through which aquatic environments and team-based water sports can support recovery and long-term survivorship outcomes.

### 4.3 Outdoor mind-body practices and forest-based activities

Mind-body disciplines such as yoga, tai chi, and qigong are increasingly recognized as supportive strategies for managing stress, anxiety, fatigue, and sleep disturbances among cancer survivors by combining gentle movement, breathing regulation, and focused attention (122). When practiced outdoor, these activities may benefit from the restorative effects of natural environments, enhancing relaxation and emotional regulation (123,124,146). Although literature remains limited, it has been demonstrated that mindful walking, outdoor yoga, or meditation-based movement in natural settings can promote relaxation, body awareness, emotional regulation, and social connection thus representing a valuable approach for BC survivors coping with the psychological aftermath of treatment (89,99,100).

Similarly, forest-based interventions, often referred to as forest bathing or Shinrin-yoku, involve immersive experiences in forest environments that emphasize sensory engagement and relaxation rather than structured exercise (125,147). Research shows that forest exposure can reduce cortisol levels, improve heart rate variability, and decrease blood pressure (73,74,126). Preliminary studies also suggest potential benefits for cancer survivors, including improvements in immune function, overall psychological wellbeing, and quality of life (80,127,128,148). These findings underpin the emerging concept of “forest medicine”, which highlights the role of forest environments in health promotion and disease prevention (34,37,125,129).

## 5 Discussion and future perspectives

The growing population of BC survivors has accelerated the transition toward survivorship-oriented models of oncology care that address long-term health, functional capacity, and psychosocial wellbeing. Within this framework, physical activity is widely recognized as a cornerstone intervention for improving survivorship outcomes (13,44,149). Nevertheless, the persistent gap between evidence-based recommendations and real-world adherence highlights the need for innovative approaches capable of enhancing engagement in exercise (20,62).

Within this context, NBPA has emerged as a promising evolution of exercise prescription in oncology by integrating physiological training with environmental context. This approach shifts the focus from exercise as a purely biomedical intervention toward a multidimensional experience shaped by ecological, psychological, and social determinants (146). From a biological perspective, NBPA may influence survivorship outcomes through complementary mechanisms. Exercise stimulates metabolic regulation, myokine release, and immune modulation, whereas nature exposure has been associated with reductions in stress-related neuroendocrine activation and improvements in autonomic balance (53,73,129). The interaction between these mechanisms suggests that physical activity performed in natural environments could amplify the therapeutic effects of exercise through synergistic psychophysiological pathways (7,53). Nevertheless, it should be acknowledged that most evidence supporting these biological interactions remains indirect. Current data do not yet allow definitive conclusions regarding whether NBPA produces superior immunological, inflammatory, or endocrine effects among BC survivors compared to conventional exercise performed in non-natural environments. The behavioral dimensions of NBPA are equally important since exercise adherence is strongly influenced by intrinsic motivation, perceived enjoyment, and environmental engagement. Natural environments may foster such factors by providing restorative and aesthetically stimulating settings that increase enjoyment and reduce perceived exertion. Outdoor group-based activities may further contribute to survivorship outcomes by fostering peer interaction through a supportive environment where movement, social connection, and exposure to nature converge to promote psychological resilience and empowerment (80,140,141). Collectively, these behavioral determinants are critical for long-term adherence, which remains a central challenge in BC survivors coping with fatigue, anxiety, and reduced confidence in physical capacity following treatment (66,86,93).

Despite these promising conceptual foundations, research on NBPA in oncology remains limited. Existing studies often involved small samples, heterogeneous intervention designs, and mixed cancer populations, hence reducing statistical power and generalizability (42,150). Additional methodological limitations need to be considered. Specifically, studies vary substantially regarding intervention duration, exercise intensity/frequency, delivery modalities, environmental characteristics, seasonality, and outcome measures. Such heterogeneity complicates comparisons across studies and limits the development of standardized recommendations. Another important consideration concerns the potential for selection bias since individuals voluntarily adhering to outdoor programs may already possess higher motivation, better baseline functional capacity, stronger social support networks, and greater affinity toward natural settings than the broader population of BC survivors. Future research should prioritize well-designed randomized controlled trials comparing NBPA with conventional exercise interventions while exploring optimal activity types, intensity, and environmental contexts. Implementation research will also be essential to concretely translate NBPA into survivorship care pathways. Community-based programs delivered in parks, forests, or aquatic environments may represent accessible and cost-effective strategies for promoting physical activity among BC survivors. However, equitable access to safe natural environments remains a challenge in many settings, highlighting the importance of interdisciplinary collaboration among healthcare professionals, public health experts, and urban planners (94,151,152). Safety and accessibility considerations represent key concepts when contemplating green prescription implementation, especially in the oncology field. Although many NBPA modalities are relatively low-cost, access to safe green or blue spaces is not uniformly distributed across geographical, socioeconomic, and urban contexts. In particular, environmental barriers may include transportation difficulties, unfavorable weather conditions, seasonal variability, limited availability of supervised programs, and concerns regarding personal safety (153). Individual factors such as treatment-related limitations, comorbidities, reduced mobility, fear of outdoor exercise, or cancer-related fatigue may further affect participation (24,25). Consequently, successful integration of NBPA into survivorship care requires individualized assessment, flexible delivery models, and attention to health equity to ensure that benefits are not restricted to specific advantaged populations.

In conclusion, as oncology continues to evolve toward integrative and patient-centered survivorship models, NBPA may represent a promising and innovative extension of exercise oncology. Current evidence suggests that combining physical activity with exposure to natural environments may support psychological wellbeing, behavioral engagement, and potentially several biological processes relevant to survivorship. However, many of the proposed mechanistic pathways remain incompletely understood and are supported primarily by indirect or preliminary evidence. Accordingly, NBPA should presently be regarded as a promising conceptual framework rather than an established clinical intervention. Future well-designed randomized controlled trials, mechanistic investigations, and implementation studies are needed to determine optimal intervention characteristics, identify target populations most likely to benefit, and clarify the comparative effectiveness of NBPA relative to conventional exercise approaches. Therefore, advancing research in this still underexplored field through interdisciplinary collaboration and the stable integration of specialized kinesiologists into evaluative and leading processes becomes essential to clarify the effectiveness of different intervention strategies and facilitate translation into clinical and community settings (27). Such a rigorous, multi-perspective approach is crucial before firm clinical recommendations can be incorporated into BC survivorship care guidelines.

## Data Availability

The original contributions presented in the study are included in the article/supplementary material, further inquiries can be directed to the corresponding author.
